# Huangqi Guizhi Wuwu decoction for diabetic peripheral neuropathy: Protocol for a systematic review

**DOI:** 10.1097/MD.0000000000016696

**Published:** 2019-08-02

**Authors:** Yuan Zhang, Guangming Gong, Xiyu Zhang, Linyue Zhou, Hongyan Xie, Yuan Tian, Chunguang Xie

**Affiliations:** Hospital of Chengdu University of Traditional Chinese Medicine, Chengdu, Sichuan Province, P.R. China.

**Keywords:** diabetic peripheral neuropathy, Huangqi Guizhi Wuwu decoction, protocol, systematic review

## Abstract

**Background:**

Diabetic peripheral neuropathy (DPN) is one of the most common causes of disability in diabetic population, and its pathogenesis is related to a variety of factors. There is currently no effective treatment for such chronic disease. Traditional Chinese medicine has a long clinical history for the prevention and treatment of diabetes and chronic complications, and it also shows certain advantages in the treatment of DPN. Many clinical studies have confirmed that Chinese medicine Huangqi Guizhi Wuwu decoction (HGWD) can reduce the clinical symptoms and improve neuronal function of patients with DPN. So we intend to conduct a systematic review further clarified the effectiveness and safety of HGWD for DPN.

**Methods:**

We will search each database from the built-in until June 2019. The English literature mainly searches Cochrane Library, PubMed, EMBASE, and Web of Science, while the Chinese literature comes from CNKI, CBM, VIP, and Wangfang database. Simultaneously we will retrieval clinical registration tests and grey literatures. This study only screen the clinical randomized controlled trials (RCTs) about HGWD for DPN to assess its efficacy and safety. The 2 researchers worked independently on literature selection, data extraction, and quality assessment. The dichotomous data is represented by relative risk (RR), and the continuous is expressed by mean difference (MD) or standard mean difference (SMD), eventually the data is synthesized using a fixed effect model (FEM) or a random effect model (REM) depending on whether or not heterogeneity exists. The clinical efficacy, median sensory nerve conduction velocity, median motor nerve conduction velocity, peroneal sensory nerve conduction velocity, and peroneal motor nerve conduction velocity were evaluated as the main outcomes. Fasting blood glucose, 2 hours postprandial blood glucose, hemorheology, and adverse reactions were secondary outcomes. Finally, meta-analysis was conducted by RevMan software version 5.3.

**Results:**

This study will synthesize and provide high-quality evidence based on the data of the currently published HGWD for the treatment of DPN, especially in terms of clinical efficacy, neurological function, blood glucose, hemorheology, and safety.

**Conclusion:**

This systematic review aims to provide new options for HGWD treatment of DPN in terms of its efficacy and safety.

**PROSPERO registration number:**

PROSPERO 2019 CRD42019132031.

## 1 Introduction

Diabetic peripheral neuropathy (DPN) is one of the most common chronic complications in the endocrinology department, and it is also a major cause of disability in diabetic patients.^[1]^ According to statistics, the prevalence of diabetes in Chinese ethnic groups is as high as 11.6%, in which the population of DPN account for nearly 50% of T1DM or T2DM.^[2,3]^ DPN mainly affect the sensory nerve and motor function, with numbness and pain as the typical clinical symptoms, mostly symmetry.^[4,5]^ The above symptoms are more severe in the lower limbs than in the upper limbs, and worsen at night and cold temperatures. Eventually, those with long duration or severe symptoms will significantly affect their quality of life.^[6,7]^

Currently, the treatment of DPN mainly includes symptomatic treatment such as controlling blood glucose level and relieving pain of neuropathy.^[8]^ In addition, many studies have confirmed that antioxidants, nutritional neuropharmaceuticals, aldose reductase inhibitors, etc can be used to treat DPN, but they are characterized by toxic side effects and poor tolerance, and their clinical efficacy has not been clearly confirmed.^[9]^ Therefore, multiple researches are needed to demonstrate an effective method for treating peripheral nerves in diabetes.

In recent years, the advantages of traditional Chinese medicine in the prevention and treatment of this kind of chronic diseases have been widely recognized around the world.^[10]^ The prescription of HGWD firstly recorded in the book of *Jingui Yaolue* written by Zhang Zhongjing, is composed of Radix Astragali (Huangqi), Ramulus Cinnamomi (Guizhi), Paeonia lactiflora (Shaoyao), Rhizoma Zingiberis Recens (Shengjiang), and Jujube (Dazao). And it has been used in the treatment of snoring for thousands of years in China. Regardless of a large number of animal experiments or clinical trials, HGWD has been shown to improve neurological function and increase blood circulation by enhancing the expression of neurotrophic factors or reducing peroxide production.^[11,12]^ Therefore, we intend to collect randomized controlled trials (RCTs) about HGWD for DPN based on the basis of evidence-based medicine, and conduct a meta-analysis of its efficacy and safety to provide higher quality clinical evidence for Chinese medicine treatment of DPN.

## 2 Methods

### 2.1 Protocol registration

The systematic review protocol has been registered on the prospero website as CRD 42019132031 (http://www.crd.york.ac.uk/PROSPERO/display_record.php?ID=CRD42019132031). It is reported following the guidelines of Cochrane Handbook for Systematic Reviews of Interventions and the Preferred Reporting Items for Systematic Reviews and Meta-analysis Protocol (PRISM).^[13]^ If there are any adjustments throughout the study, we will fix and update the details in the final report.

### 2.2 Inclusion criteria

#### 2.2.1 Study design

The study only select clinical randomized controlled trials of HGWD for DPN published in both Chinese and English. However, animal experiments, reviews, case reports, and non-randomized controlled trials are excluded.

#### 2.2.2 Participants

The patients of DPN must meet the diagnostic criteria established by the 2016 Chinese Medical Association Diabetes Branch, regardless of race, sex, and age.^[14]^ Neuropathy caused by other causes and patients with severe heart disease, liver and kidney dysfunction, mental illness, or a relevant drug allergic history will be not included.

#### 2.2.3 Interventions

Both groups were cured with conventional diabetes treatments recommended by the ADA guidelines, including diet, exercise, and hypoglycemic, and lipid-lowering therapies.^[15]^ The experiment group used HGWD or modified HGWD, while the control group applied for placebo, nutritional neurological drugs, or no treatment. In addition, the 2 groups did not take any drugs that interfered with the outcome indicators. The follow-up time was ≥4 weeks.

#### 2.2.4 Outcomes

The primary outcomes include the improvement in clinical efficacy and nerve conduction velocity. The clinical efficacy refers to the guiding principles for clinical research of new Chinese medicines^[16]^ and is determined according to the degree of improvement of the symptoms of the patient before and after treatment: markedly effective: symptoms improved significantly >70%; effective: symptoms reduced by 30% to 70%; ineffective: symptom improvement is <30% or no improvement, or even worse. The nerve conduction velocity includes the sensory nerve conduction velocity and the motor nerve conduction velocity, which are evaluated by electromyography.

Secondary outcomes are mainly composed of fasting blood glucose, 2 hours postprandial blood glucose, hemorheology (blood viscosity, fibrinogen concentration), and adverse events.

### 2.3 Search methods

#### 2.3.1 Electronic searches

We will retrieve each database from the built-in until June 2019. The English literature mainly searches Cochrane Library, PubMed, EMBASE, and Web of Science. While the Chinese literature comes from CNKI, CBM, VIP, and Wangfang database. We adopt the combination of heading terms and free words as search strategy which decided by all the reviewers. Search terms: Huangqi Guizhi Wuwu Tang, Huangqi Guizhi Wuwu Decoction, Huang Qi Gui Zhi Wu Wu, Huangqi Guizhi Wuwu, diabetes, diabetic, diabetes mellitus, diabetic peripheral neuropathy, diabetic neuropathies. We will simply present the search process of the Cochrane library (Table 1). Adjusting different search methods according to different Chinese and English databases.

**Table 1:**
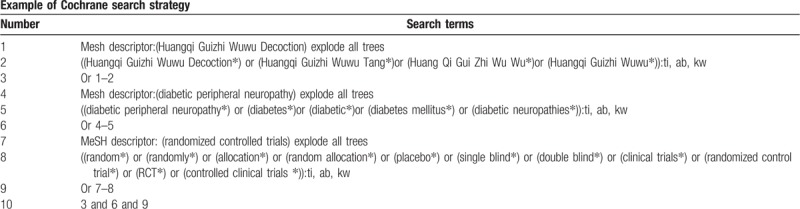
Cochrane library search strategy.

#### 2.3.2 Searching other resources

At the same time, we will retrieve other resources to complete the deficiencies of the electronic databases, mainly searching for the clinical trial registries and grey literature about HGWD for DPN on the corresponding website.

### 2.4 Data collection and analysis

#### 2.4.1 Selection of studies

**Figure 1:**
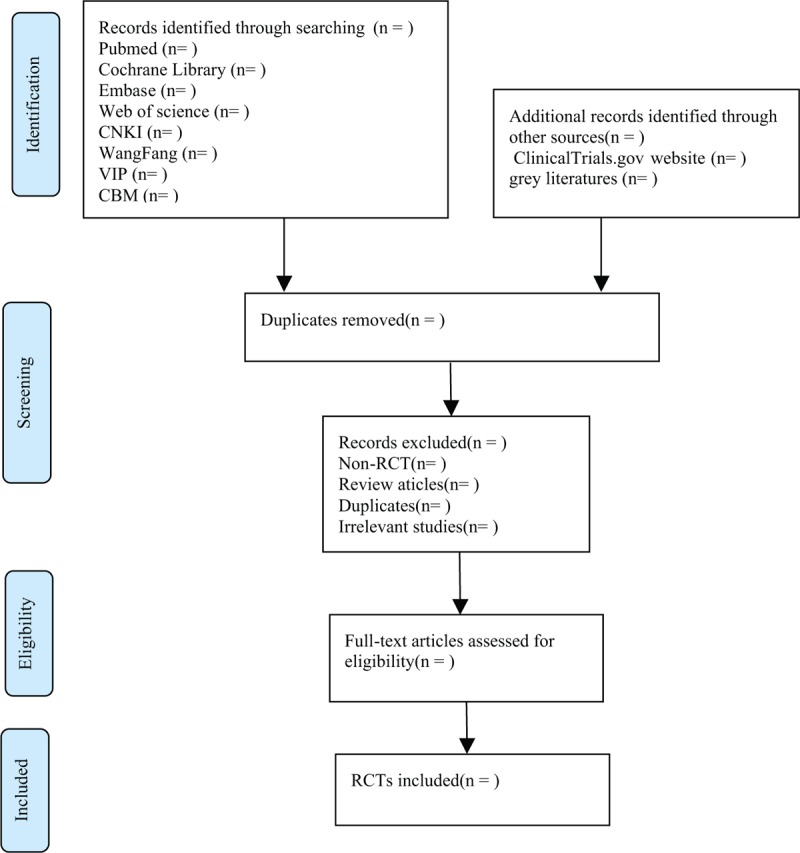
Flow chart of the study selection.

Import all literatures that meet the requirements into Endnote X8 software (Thomson Research Soft, Stanford, Connecticut). First of all, 2 independent reviewers initially screened the literatures that did not meet the pre-established standards of the study by reading the title and abstract. Secondly, download the remaining literatures and read the full text carefully to further decide whether to include or not. Finally, the results were cross-checked repeatedly by reviewers. If there is a disagreement in the above process, we can reach an agreement by discussing between both reviewers or seek a third party's opinion. Flow chart of the study selection (Fig. 1) will be used to show the screening process of the study.

#### 2.4.2 Data extraction and management

According to the characteristics of the study, we prepare an excel form for data collection before data extraction. Outcome indicators for eligible studies were independently extracted and filled in the data extraction form by 2 reviewers. If there is any argument, it can get an agreement by discussing through 2 reviewers or seek a third party's suggestion. The main data extracted are as follows: title, author, year, fund source, sample size, age, sex, duration of disease, interventions, outcome measures, adverse reactions, etc. If you find something unclear in the study, you can contact the author of the communication directly for more detailed information. The above information was finally cross-checked by 2 reviewers.

#### 2.4.3 Assessment of risk of bias in included studies

The quality assessment of RCTs adopts the risk of bias (ROB) assessment tool provided by the Cochrane Handbook. The following 7 items, such as random sequence generation, allocation concealment, blinding of participants and personnel, blinding of outcome assessment, incomplete outcome data, selective outcome reporting, and other bias, are evaluated by 3 grades of “low bias,” “high bias,” and “unclear bias.” The discrepancies will get a consistent conclusion by discussing between both reviewers or seeking the third-party consultation.

#### 2.4.4 Measures of treatment effect

Different evaluation methods are selected according to the different efficacy indicators. For the dichotomous data, we will choose the effect scale indicator relative risk (RR) with 95% confidence interval (CI) to represent. While the continuous data is expressed as mean difference (MD) or standardized mean difference (SMD) with 95% CI depending on whether the measurement scale is consistent or not.

#### 2.4.5 Dealing with missing data

The reviewers will contact the first author or correspondent author via email or telephone to obtain missing data if the relevant data is incomplete. If the missing data is still not obtained in the above way, we can synthesize the available data in the initial analysis. Furthermore, sensitivity analysis will be used to assess the potential impact of missing data on the overall results of the study.

#### 2.4.6 Assessment of heterogeneity

Heterogeneity will be assessed by chi-squared test and *I*^2^ test. If *I*^2^ < 50%, *P* > .1, we consider that no statistical heterogeneity between each studies and choose fixed effect model (FEM) to synthesize the data. If *I*^2^ ≥ 50%, *P* < .1, indicating that there is a statistical heterogeneity, the data are integrated by the random effect model (REM). In addition, due to differences in heterogeneity, we will conduct subgroup or sensitivity analysis to look for the potential causes.

#### 2.4.7 Data analysis

Review Manager software version 5.3 (The Nordic Cochrane Center, The Cochrane Collaboration, 2014, Copenhagen, Denmark) provided by the Cochrane Collaboration will be performed for data synthesis and analysis. The dichotomous data is represented by RR, continuous data is expressed by MD or SMD. If there is no heterogeneity (*I*^2^ < 50%, *P* > .1), the data are synthesized using a fixed effect model. Otherwise (*I*^2^ ≥ 50%, *P* < .1), a random effect model is used to analyze. Then subgroup analysis will be conducted basing on the different causes of heterogeneity. If a meta-analysis cannot be performed, it will be replaced by a general descriptive analysis.

#### 2.4.8 Subgroup analysis

If the results of the study are heterogeneous, we will conduct a subgroup analysis for different reasons. Heterogeneity is manifested in the following several aspects, such as race, age, sex, different intervention forms, pharmaceutical dosage form, dosage, treatment course.

#### 2.4.9 Sensitivity analysis

Sensitivity analysis is mainly used to evaluate the robustness of the primary outcome measures. The method is that removing the low-level quality study one by one and then merge the data to assess the impact of sample size, study quality, statistical method, and missing data on results of meta-analysis.

#### 2.4.10 Reporting bias

If there are >10 studies in the meta-analysis, the symmetry of the funnel plot will be assessed to examine publication bias, with results being interpreted cautiously.

#### 2.4.11 Grading the quality of evidence

In this systematic review, the quality of evidence for the entire study is assessed using the “Grades of Recommendations Assessment, Development and Evaluation (GRADE)” standard established by the World Health Organization and international organizations.^[17]^ To achieve transparency and simplification, the GRADE system divides the quality of evidence into 4 levels: high, medium, low, and very low. The GRADE profiler 3.2 will be employed for analysis.

## 3 Discussion

DPN is a common chronic complication of diabetes.^[18]^ At present, drug treatment is universally used in the treatment of this disease, and there are some shortcomings, such as little effect or large side effects. Therefore, both clinicians and patients hope to seek a new treatment to improve symptoms with low adverse reactions.

Traditional Chinese medicine has been used to treat diabetes and diabetic complications in China for many years.^[19,20]^ It has not only outstanding efficacy, but also has few side effects and low economic costs. Clinical studies have shown that HGWD can alleviate the symptoms of DPN and improve the overall clinical efficacy.^[21–23]^

However, there is no evidence-based medicine to confirm the efficacy of HGWD for DPN. So we attempt to perform this meta-analysis to provide high-quality evidence for the clinical efficacy and safety of HGWD.

Similarly, we hope this study will provide more options for clinicians and patients to treat DPN. There may be some potential deficiencies in this systematic review. Firstly, both Chinese and English forms of research may increase the bias of the study. Secondly, the variety of race, age, sex, intervention forms, pharmaceutical dosage form, dosage, and treatment course may result in higher clinical and statistical heterogeneity. Finally, the clinical trial design of traditional Chinese medicine is difficult to achieve single or double blind method throughout all the study.

## Author contributions

**Conceptualization:** Yuan Zhang, Guangming Gong, Chunguang Xie.

**Data curation:** Xiyu Zhang, Yuan Tian.

**Formal analysis:** Linyue Zhou, Yuan Tian.

**Funding acquisition:** Chunguang Xie.

**Methodology:** Xiyu Zhang, Hongyan Xie.

**Project administration**: Chunguang Xie, Guangming Gong.

**Resources:** Yuan Zhang, Guangming Gong.

**Software:** Yuan Zhang.

**Supervision:** Hongyan Xie

**Writing – original draft:** Yuan Zhang, Guangming Gong.

**Writing – review & editing:** Chunguang Xie.
